# Diffusion Tensor Tractography Studies of Central Post-stroke Pain Due to the Spinothalamic Tract Injury: A Mini-Review

**DOI:** 10.3389/fneur.2019.00787

**Published:** 2019-08-02

**Authors:** Sung Ho Jang, Jeong Pyo Seo, Sung Jun Lee

**Affiliations:** Department of Physical Medicine and Rehabilitation, College of Medicine, Yeungnam University, Daegu, South Korea

**Keywords:** central post-stroke pain, stroke, spinothalamic tract, diffusion tensor imaging, diffusion tensor tractography

## Abstract

Elucidation of the pathophysiological mechanism of central post-stroke pain (CPSP) is essential to the development of effective therapeutic modalities for CPSP. However, the pathophysiological mechanism of CPSP has not yet been clearly elucidated. The recent development of diffusion tensor tractography (DTT), derived from diffusion tensor imaging (DTI), has allowed visualization and estimation of the spinothalamic tract (STT), which has been considered the most plausible neural tract responsible for the pathogenesis of CPSP. In this mini-review, six DTT studies in which CPSP due to STT injury in stroke patients was demonstrated are reviewed. The information provided in the reviewed studies suggests that DTT is useful in the elucidation of the pathophysiological mechanism associated with CPSP. We believe that the reviewed studies will facilitate neurorehabilitation of stroke patients with CPSP. However, DTT studies of CPSP are still in the beginning stage because the total number (six studies) of the reviewed studies is very low and half were case reports. Therefore, further studies involving large numbers of subjects are warranted.

## Introduction

Stroke is a leading cause of mortality and morbidity in adults. Central post-stroke pain (CPSP) is a neuropathic pain caused by cerebrovascular insult to the somatosensory pathway in the brain (1). CPSP, which is one of the most common and annoying sequelae following stroke, has been reported by up to 12% of stroke patients and is related to poor quality of life after stroke (2–6). Despite the high prevalence of CPSP and its negative impact on quality of life in stroke patients, there has been no specific therapeutic modality for complete cure of CPSP (3, 7, 8). As a result, a significant portion of stroke patients with CPSP are obliged to take large amounts of medicine for a long period of time or suffer intractable CPSP.

For management of CPSP, medications including antidepressants, antiepileptic drugs, and opioids have commonly been used for a long time (3, 9). Recently, neuromodulations including deep brain stimulation, repetitive transcranial magnetic stimulation (rTMS) and transcranial direct current stimulation (tDCS) have become management modalities for patients with CPSP (1, 10–15). However, the methods for application of neuromodulations have not been definitively settled upon and are controversial in some aspects (16). This appears to be related to the pathophysiological mechanism of CPSP not being clearly elucidated yet, even though clarification of the pathophysiological mechanism of CPSP is mandatory for development of effective therapeutic modalities for management of CPSP (3, 17–23).

Several theories including central sensitization, neural excitability changes due to disinhibition, alteration of the somatosensory function, changes in thalamic function, and inflammation of an involved neural tract have been suggested as pathophysiological mechanisms for CPSP (3, 17, 20, 21, 23–26). Several neural structures, including the spinothalamic tract (STT), medial lemniscus pathway (MLP), thalamus, and cerebral cortex, have been suggested as being responsible for the pathogenesis of CPSP (3, 17, 20, 21, 23–28). Recently, the STT has been considered the most plausible neural tract responsible for the pathogenesis of CPSP (3, 17, 19, 20, 23, 24, 29–31).

Precise evaluation of the STT had been impossible because it cannot be clearly identified from adjacent neural structures by conventional brain magnetic resonance imaging. In contrast, the recent development of diffusion tensor tractography (DTT), derived from diffusion tensor imaging (DTI), allows us to visualize and estimate the STT three dimensionally at the subcortical level (22). Therefore, many studies have demonstrated STT injury in terms of configuration and changes of DTT parameters in patients with brain injury (19, 23, 24, 29–33).

**Figure 1:**
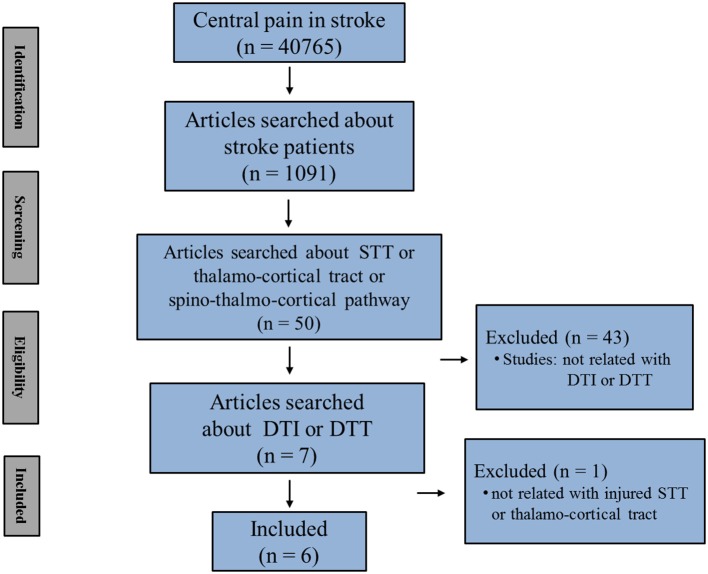
Flow diagram of the study selection process (STT, spinothalamic tract; DTI, diffusion tensor imaging; DTT, diffusion tensor tractography).

In this mini-review, DTT studies that demonstrated CPSP because of STT injury in stroke patients are reviewed. Relevant studies in the period from 1990 to 2018 were identified using the following electronic databases: PubMed, Google Scholar, and MEDLINE. Additionally, the following keywords/abbreviations were used to search the databases: DTI, DTT, CPSP, STT, thalamocortical tract, spino-thalamo-cortical pathway, stroke, intracerebral hemorrhage (ICH), and cerebral infarct. This review was limited to studies of humans with stroke. We selected relevant studies according to the flow diagram shown in Figure 1. Overall, six studies were selected and reviewed (Table 1) (19, 23, 24, 29–31).

**Table 1:** Diffusion tensor tractography studies of central post-stroke pain caused by the spinothalamic tract injury.

**Authors**	**Publication year**	**Number of patients**	**Duration to DTI**	**Pathology and lesion location**	**DTT analysis method and results**	**Limitations**
Seghier et al. (23)	2005	1	4–5 years	Intracerebral hemorrhage (thalamus and internal capsule)	Configuration (fiber density↓)	Uncertain ROIs, case report, no reliability for DTT
Goto et al. (19)	2008	17	5.1 years (1–8.8 years)	Stroke (supratentorial area)	Higher delineration ratio: rTMS effective	Not pure STT, no reliability for DTT
Hong et al. (24)	2010	30	20 months (5–48 months)	Intracerebral hemorrhage (corona radiata and basal ganglia)	Laterality index of DTT parameters (FA, MD, TV↓)	No reliability for DTT
Hong et al. (29)	2012	52	18.8 months (5–46 months)	Intracerebral hemorrhage (corona radiata, basal ganglia, and thalamus)	Configuration (impaired integrity), DTT parameters (FA, MD, TV↓)	Simple analysis using integrity of STT, no reliability for DTT
Jang et al. (30)	2017	5	11 days (10–13 days)	Cerebral infarct (corona radiata, thalamus, and pre- and post-central gyrus)	DTT parameters (FA↓, MD↑, TV↓)	Case series, no reliability for DTT
Jang et al. (31)	2018	1	2 weeks 14 months	Intracerebral hemorrhage (thalamus)	Configuration (partial tearing and thinning)	Case report, no reliability for DTT

*DTT, diffusion tensor tractography; FA, fractional anisotropy; MD, mean diffusivity; TV, tract volume; rTMS, repetitive transcranial magnetic stimulation; ROIs, regions of interest; STT, spinothalamic tract*.

## Diffusion Tensor Tractography for the Spinothalamic Tract

DTT for reconstruction of the neural tracts usually employs a combined region of interest (ROI) method that reconstructs only neural fibers passing more than two ROI areas. The ROI areas and reconstruction conditions for the neural tracts are well-defined for each neural tract (34–36). Many studies have demonstrated excellent reliability of DTT method for various neural tracts (34–36). However, the reliability of DTT for the STT has not demonstrated.

The main advantage of DTT over DTI is that the entire neural tract can be evaluated in terms of DTT parameters. Among the various DTT parameters, fractional anisotropy (FA), mean diffusivity (MD), and tract volume (TV) are most commonly used to evaluate the state of the STT in patients with brain injury (24, 29, 30, 33, 34, 37, 38). The FA, which indicates the degree of directionality of water diffusion, is used to assess the degree of directionality and integrity of white matter microstructures such as axons, myelin, and microtubules (34, 37, 38). The MD indicates the degree of water diffusion, which increases in some pathologies such as vasogenic edema or axonal damage (34, 37, 38). In contrast, the TV suggests the number of voxels that are included in a neural tract (34, 37, 38). Therefore, decreases in the values of the FA and TV, and increases in the value of the MD of the STT indicate neural injury of the STT.

However, several limitations of DTT should be considered (39–41). First, the fiber tracking technique is operator-dependent. Second, DTT may underestimate the fiber tracts. DTT is a powerful anatomic imaging tool that can demonstrate the gross fiber architecture, but not the functional or synaptic connections. Third, regions of fiber complexity and crossing can prevent full reflection of the underlying fiber architecture by DTT (39–41).

## Studies of Central Post-Stroke Pain Due to Spinothalamic Tract Injury That was Demonstrated on DTT

The first study after introduction of DTI was reported by Seghier et al. (23). A 65-year old man had suffered a hypertensive ICH developed in his left side 4 months after onset and became aggravated during winter at ~4 years after onset. Brain MRI conducted ~4 years after onset showed a residual hemorrhagic cavity in the right ventral posterolateral nucleus of the right thalamus and the adjacent posterior third of the posterior limb of the internal capsule. DTIs taken 6 months after brain MRI; ROIs were placed manually on the FA map and continuous tracking method (streamline-like approach) was used for reconstruction method for DTT. DTT results revealed a reduction in fiber density of the lateral thalamocortical tact, which was presumed to be the STT, whereas the spinothalamic pathway below the thalamus and the medial thalamocortical fibers, which were presumed to be the MLP, were spared. Functional MRI performed using different thermonociceptive stimuli showed pain-specific signal changes in the anterior cingulate gyrus and the parietal regions. As a result, the authors concluded that injury of the lateral nociceptive thalamocortical tract with release of activity of the anterior cingulate and posterior parietal regions appeared to lead to the pathogenesis of CPSP in this patient (23). However, because the locations of the ROIs were not described in detail, it is uncertain whether the reconstructed lateral thalamocortical tract purely reflected the STT, although it was presumed to be the STT. Furthermore, this study was limited as it was a single case report.

Subsequently, Goto et al. (19) investigated the effects of rTMS for CPSP that was refractory to medications in stroke patients with supratentorial lesions (19). They reconstructed the corticospinal tract (seed ROI: cerebral peduncle; target ROI: precentral gyrus) and the thalamocortical tract (seed ROI: cerebral peduncle; target ROI: postcentral gyrus), which was presumed to be the combined STT and MLP, in 13 of 17 chronic stroke patients with CPSP using Volume-One and dTV software (free software by Masutani). They found lesions in the thalamocortical tract of the affected hemisphere in all 13 patients who their thalamocortical tract reconstructed. They applied rTMS to the primary motor cortex (90% intensity of the resting motor threshold, intensity: 100 A/us, 10 trains of 10-s, 5-Hz TMS pulses with 50 s intervals between trains) and classified the patients into an rTMS-effective group (more than 30% decrease in Visual Analog Scale [VAS] score, eight patients) and an rTMS-ineffective group, nine patients). They then estimated relationships between the delineation ratio (defined as the ratio of the cross section of the affected side to that of the unaffected side) of the corticospinal and thalamocortical tracts and the effect of rTMS on CPSP. The rTMS-effective group showed higher delineation ratios for the corticospinal tract and the thalamocortical tract than the rTMS-ineffective group. In other words, the patients who had more remaining neural fibers of the corticospinal tract and the thalamocortical tract in the affected hemisphere were able to relieve the intensity of CPSP. As a result, the authors suggested that the thalamocortical tract also plays a role in pain reduction by rTMS on the primary motor cortex and that the efficacy of rTMS for patients with CPSP is predictable by DTT (19). However, the thalamocortical tract appeared not to reflect the pure STT considering that the ROIs were selected for reconstruction of the thalamocortical tract.

In 2010, Hong et al. investigated the relationship between STT injury and CPSP in patients with ICH (hematoma confined within the corona radiata and basal ganglia) using DTT for the STT and MLP (24). Thirty consecutive chronic patients (average 20 months after onset) in whom integrity of the STT and MLP were spared in both hemispheres were recruited. They classified the patients into the CPSP group (16 patients) and the non-CPSP group (14 patients) according to the presence of the CPSP. The STT and MLP were reconstructed using two ROIs (The seed ROI: the STT-the STT area of the posterolateral medulla and the MLP: the MLP area of the anteromedial medulla, and the target ROI: the primary somatosensory cortex). Fiber tracking was performed using a probabilistic tractography method based on a multifiber model (the Oxford Center for Functional Magnetic Resonance Imaging of the Brain Software Library [FSL; www.fmrib.ox.ac.uk/fsl]). The laterality index (the value of a tract in the affected hemisphere—the value of a tract in the unaffected hemisphere)/(the value of a tract in the affected hemisphere + the value of a tract in the unaffected hemisphere) was estimated to determine the asymmetry of DTT parameters between hemispheres. Only the laterality index for the TV of the STT in the CPSP group was lower than that of the non-CPSP group without differences in other DTT parameters (the FA and MD values). The decrease in TV without changes in the FA or MD values in the CPSP group indicates partial injury of the STT. Therefore, the authors concluded that partial injury of the STT appeared to be a requirement for the development of CPSP in patients with ICH (24). The reliability in terms of the repeatability and reproducibility for the reconstruction method of the STT was not demonstrated.

In 2012, Hong et al. investigated the prevalence of CPSP according to the integrity of the STT in patients with ICH (hematomas confined within the corona radiata, basal ganglia, or thalamus) (29). They recruited 52 chronic patients (average 18.8 months after onset) and reconstructed the STT using two ROIs (seed ROI: the STT area of the posterolateral medulla, which was posterior to the inferior olivary nucleus, anterior to the inferior cerebellar peduncle, and lateral to the medial lemniscus; target ROI: the ventral posterior lateral nucleus of the thalamus). The patients were classified into two groups according to preservation of the integrity of the STT (the preserved group and the interrupted group), and each group was divided into two subgroups according to the presence of CPSP (the CPSP subgroup and the non-CPSP subgroup). The preserved group included 34 patients (the CPSP subgroup, 16 [47%] patients; the non-CPSP subgroup, 18 [53%] patients), and 18 patients were enrolled in the disrupted group (the CPSP subgroup, 3 [17%] patients; the non-CPSP subgroup, 15 [83%] patients). The prevalence of CPSP in the preserved group (CPSP, 16 patients; non-CPSP, 18 patients) was higher than that in the disrupted group (CPSP, 3; non-CPSP, 15). The TV of the CPSP subgroup in the preserved group was lower than those in both the non-CPSP subgroup and the control group. Neither FA nor MD values showed significant differences between the CPSP and non-CPSP subgroups of the preserved group. As a result, the authors found that the prevalence of CPSP in patients with partial injury of the STT was higher than that of patients with complete injury of the STT and concluded that partial injury of the STT appears to be more vulnerable to development of CPSP than complete injury of the STT in patients with ICH (29). The reliability of the reconstruction method for the STT was not described. In addition, the incidence of CPSP was classified only according to the integrity of the STT.

**Figure 2:**
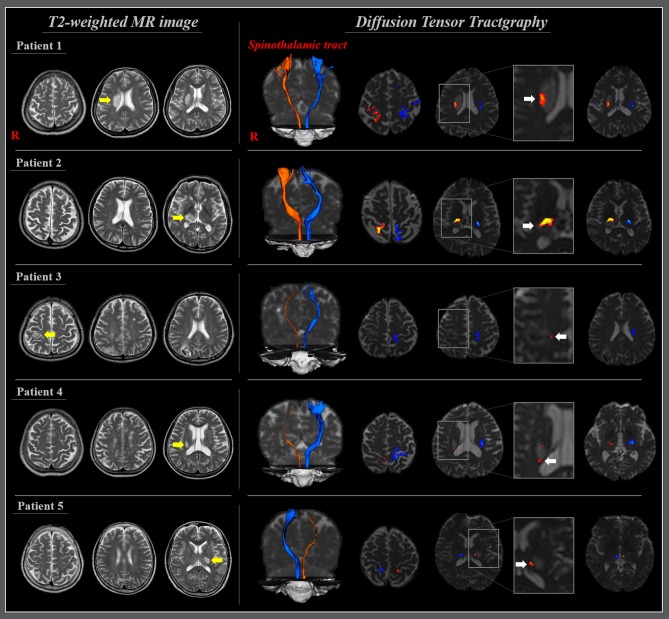
T2-weighted MRI and diffusion tensor tractography of patients with CPSP following cerebral infarction. T2-weighted MR images of five patients with cerebral infarction (yellow arrows). Diffusion tensor tractography of five patients at 11 days on average after stroke onset; all the reconstructed spinothalamic tracts in the affected hemisphere originated from the posterolateral medulla and terminated at the primary somatosensory cortex through adjacent part of the infarct (white arrows) and narrowing in three patients (patients 3, 4, and 5) [reprinted with permission from Jang et al. (30)].

During the same year, Jang et al. (30) investigated the STT injury in patients with CPSP following cerebral infarct (30). Five patients with CPSP following cerebral infarct (two patients, corona radiata; two patients, thalamus; one patient, pre-and post-central gyri; average 11 days after onset) were recruited. The STTs were reconstructed using two ROIs methods (seed ROI, the STT area of the posterolateral medulla; target ROI, the primary somatosensory cortex). Abnormal STT was detected on configuration (narrowing: three patients) or changes of DTT parameters of the STT in the affected hemisphere. Specifically, the values of the FA and TV were decreased by more than two standard deviations in two (patient 1 and 2) and three (patient 3, 4, and 5) patients, respectively, compared with those of the normal control subjects (Figure 2). In contrast, the MD value was increased by more than two standard deviations in one patient (patient 2). As a result, they demonstrated STT injury in the affected hemisphere in five patients who showed CPSP following cerebral infarct and concluded that the STT injury is a pathophysiological mechanism of CPSP in patients with cerebral infarct (30). This study was limited as it was a case series including five patients; moreover, the reliability of the reconstruction method for the STT was not clarified.

Recently, Jang et al. (31) reported on a patient who showed delayed onset CPSP because of degeneration of the STT (31). A 57-year-old female presented with right hemiparesis that occurred at the onset of a spontaneous left thalamic hemorrhage. Approximately 6 months after onset, she began to experience pain in the right arm and leg that slowly intensified with time. At 14 months after onset, the characteristics and severity of her pain were assessed as follows: continuous pain without allodynia or hyperalgesia; tingling and cold-sensational pain in her right whole arm and leg (VAS score: 5). No evidence of peripheral neuropathy or radiculopathy was detected on electromyographic assessment. For reconstruction of the STT, the seed ROI was placed on the STT area in the posterolateral medulla and the target ROI was placed on the primary somatosensory cortex. The STT configuration was well-preserved in both hemispheres on 2-week DTT, whereas the left STT, which passed through the vicinity of the thalamic lesion, showed partial tearing and thinning on 14-month DTT. As a result, the authors concluded that the STT degeneration in the affected hemisphere was demonstrated using serial DTTs in a patient with delayed onset CPSP following thalamic hemorrhage (31). However, this study was limited because it was a single case report and was without DTT parameter data.

## Conclusions

In this mini-review article, six DTT studies of CPSP related to STT injury in stroke patients were reviewed. The information provided in these reviewed studies suggests that the STT injury is an important pathophysiological mechanism of CPSP and that DTT is useful for elucidation of the pathophysiological mechanism associated with CPSP in stroke patients. However, only six studies on this topic have been reported to date, half of which were case reports. In addition, the two studies at the beginning of the series did not appear to reconstruct the STT precisely; moreover, reliability in terms of the repeatability and reproducibility of the reconstruction method for the reconstructed neural tracts was not demonstrated (19, 23). Therefore, further studies involving large numbers of subjects and using a precise reconstruction method for the STT, including assessment of reliability, are warranted. The reviewed studies also recruited patients with wide-ranging lesion locations (mainly, supratentorial lesion). Because CPSP might be different according to lesion location in terms of incidence, intensity, and characteristics, further studies with detailed information on lesion location such as thalamus and brainstem are needed. Moreover, further studies of other neural tracts such as the MLP, which is known to be involved in the pathogenesis of CPSP, are warranted (27, 28). We believe that the reviewed studies and further studies will be helpful in understanding the characteristics of CPSP according to lesion location and will facilitate precise neurorehabilitation of stroke patients with CPSP if they provide information regarding the proper application of neuromodulation, which has been applied to relieve CPSP in recent years.

## Author Contributions

SJ: study concept and design, manuscript development, writing, and funding. JS: study support. SL: critical revision of manuscript for intellectual content.

### Conflict of Interest Statement

The authors declare that the research was conducted in the absence of any commercial or financial relationships that could be construed as a potential conflict of interest.
